# Low haemoglobin in arduous seasons is associated with reduced chance of ovulation among women living in the Bolivian *altiplano*

**DOI:** 10.1093/emph/eoae022

**Published:** 2024-09-12

**Authors:** Rose Stevens, Alexandra Alvergne, Virginia J Vitzthum

**Affiliations:** School of Anthropology and Museum Ethnography, The University of Oxford, Oxford, OX2 6PE, UK; School of Anthropology and Museum Ethnography, The University of Oxford, Oxford, OX2 6PE, UK; ISEM, Univ Montpellier, CNRS, IRD, Montpellier, France; Department of Medicine, CEMCOR, University of British Columbia, CanadaV5Z 1M9; Department of Anthropology, Indiana University, Bloomington, IN 47405, USA

**Keywords:** haemoglobin, iron, anaemia, ovulation, seasonal ecology, life history trade-offs, longitudinal cohort study

## Abstract

**Background and objectives:**

Female reproductive function flexibly responds to ecological variation in energy availability, but the roles of other ecologically limited resources, such as iron, remain poorly understood. This analysis investigates whether haemoglobin associates with investment in reproductive function in a rural natural fertility population living in the Bolivian *altiplano*.

**Methodology:**

We conducted a cross-sectional secondary analysis of prospectively collected biomarker and sociodemographic data, comprising 152 menstrual cycles from 96 non-contracepting women living at 3800 m altitude. Multivariable multilevel models were used to investigate (i) whether haemoglobin concentration is associated with ecological variation in subsistence strategy and seasonal conditions, and (ii) whether haemoglobin concentration is associated with the occurrence of ovulation and/or the concentration of luteal progesterone, two biomarkers of current investment in reproduction.

**Results:**

Haemoglobin concentrations were lower in arduous seasons among those women more dependent on traditional agropastoral subsistence strategies (*β* = −0.42, 95% CI: −0.80 to −0.04, *P* = 0.032). During more arduous seasons, a 1 standard deviation increase in haemoglobin was associated with an over 3-fold increase in the odds of ovulation after adjusting for body fat, breastfeeding status, and age (adjusted odds ratio = 3.27, 95% CI: 1.10 to 9.27, *P* = 0.033).

**Conclusions and implications:**

When conditions are relatively harsh and may be expected to improve, low haemoglobin levels are associated with lower current investment in reproduction and reduced fecundity. These results support the role of iron, independent of energy stores, as a limiting resource in modulating reproductive trade-offs.

## 1. INTRODUCTION

Iron is recognized as an essential nutrient, yet anaemia remains a major global health problem affecting nearly one-third of all non-pregnant women [1]. Maternal anaemia increases the risks of poor pregnancy outcomes including maternal and offspring death [2]. Various intervention efforts to reduce maternal anaemia have not been as successful as expected, often for unknown reasons [3]. Intervention strategies to meet the needs of women with maternal anaemia require a better understanding of the intricate connections between iron status and the underlying biological mechanisms governing fecundity. Here we use a reproductive ecology approach [4] and prospective physiological data from a traditional non-contracepting high fertility population to shed new light on the socio-ecological determinants of variation in iron status in women and the consequences of such variation for fecundity across socio-ecological contexts.

Iron, a limited resource [5] tightly regulated within the body (Box 1), is critical for both successful reproduction and maternal health. An offspring’s survival requires that a mother transfers sufficient iron to the foetus during gestation, particularly as iron concentration in human milk is low. This substantial transfer of iron from mother to foetus throughout pregnancy depletes maternal iron stores [6], leaving women at elevated risk of anaemia during pregnancy. Maternal anaemia and low haemoglobin prior to and during pregnancy are associated with a host of negative outcomes for mother and baby [7], including preterm birth, poor maternal and foetal weight gain, low birth weight, growth faltering, and obstetric complications. For instance, Gambian women with lower haemoglobin levels (defined as <12 g/dl) during the two years prior to giving birth had a greater risk of stillbirths and higher mortality rates amongst their live-born children [8]. Not only is iron associated with post-conception outcomes, it may be associated with risk of conception itself. In the same Gambian population, women with low haemoglobin levels prior to pregnancy also had longer interbirth-intervals [9]. In a Polish agricultural population, higher haemoglobin was associated with a thicker endometrium, which may increase the chance of successful implantation [10]. However, the relationship between fertility and iron may be U-shaped as women with the highest haemoglobin levels among the Tsimane in Bolivia showed a reduced chance of fertility across the study period [11]. There is little understanding of the mechanisms underlying the observed associations between iron and fecundity. Studies in non-clinical settings are rare, and the lack of a theoretical framework for understanding normal variation in reproductive outcomes in response to iron biology hinders our ability to inform interventions.

Box 1: **Iron in the body**

Mechanistically, iron is a crucial resource for many biological functions, as many fundamental bodily processes evolved during a period when available iron was abundant in the environment [22]. Iron is predominantly used for synthesizing red blood cells, within which it plays a critical role in the binding of oxygen to haemoglobin for transportation around the body. Iron also has critical roles in enzymes used in respiration, as well as uses in cellular metabolism, survival, and proliferation. Iron is a particularly important bodily resource at high altitudes, where decreased oxygen availability requires physiological adjustments to allow for enough oxygen to reach the body’s tissues [22]. In Andean populations, this adjustment is achieved through the mechanism of increased haemoglobin concentration among high-altitude residents [23] and as such cut offs for defining anaemia are higher in these populations [24].

Iron levels and storage must be carefully regulated as there is no active process that eliminates iron from the body and excess bioavailable iron can cause oxidative damage or can be used by pathogens for increased proliferation. Thus, most of the ~4–5 g of iron in the body is stored and its release is tightly regulated. Iron is typically stored as ferritin and transferrin, levels of which are controlled by hepcidin, a hormone that regulates absorption of iron from the gut and release of iron stores [23]. Thus, hepcidin controls how much iron is available as haemoglobin, which is the protein that carries oxygen in red blood cells and is the indicator used to measure anaemia status.

Low haemoglobin levels can be due to, amongst other things, (i) iron-deficiency anaemia, caused by nutritional deficiencies or blood loss, and (ii) anaemia of infection, caused by immune stress. While low haemoglobin indicates low bioavailability of iron, whether this represents low iron stores also depends on the type of anaemia. Iron deficiency anaemia is associated with low total iron stores (as identified through low haemoglobin and low ferritin). Anaemia of infection is associated with normal to high total iron stores (low haemoglobin and high ferritin), but in this case, iron is withheld from the blood, particularly when inflammation is high, to avoid utilization of iron by the infecting pathogens. Haemoglobin is one of the last indicators to drop in the case of iron deficiency, so low haemoglobin can either point to very low iron stores or the presence of an infection/high inflammation [7].

At the physiological level, reproductive ecology studies suggest that iron may regulate resource allocation trade-offs between current vs. future reproduction by influencing key processes such as energy metabolism, oxidative stress, and immune function [7]. Reproductive ecology research draws on life history theory [12, 13], a branch of evolutionary biology positing that organisms have limited resources for fulfilling the basic demands of reproduction, growth, and survival, leading to resource allocation trade-offs between essential fitness functions. While decades of human reproductive ecology research have shown that energy availability modulates reproductive trade-offs across a range of ecological settings [14–17], the importance of other limited resources, iron being a key candidate given its association with reproductive outcomes, remains under-researched [18]. That iron is involved in modulating the trade-off between current and future reproductive investment is suggested by a study in northern Kenya, where higher haemoglobin was associated with a greater chance of having resumed menstrual periods post-birth [19]. Iron has been shown to be necessary for the function of granulosa cells, which make up the follicle surrounding a maturing egg [20]. With reduced functioning of these cells, the ovaries will produce lower oestrogen levels [21], the chances of ovulation may decrease, and subsequent progesterone levels and endometrial growth would be lower. However, how iron modulates ovulation and levels of luteal progesterone, two markers of fecundity shaped by energy-dependent reproductive trade-offs across a range of ecological settings [4], is currently not well understood.

In this paper, we investigated the relationship between iron and markers of fecundity using prospective data on haemoglobin and progesterone levels from a unique natural fertility sample of Bolivian women. Our objectives are two-fold (i) to investigate if haemoglobin levels co-vary with women’s socio-ecology, focussing on the influence of seasons and variation in wealth and subsistence strategies, and (ii) to investigate if variation in fecundity, specifically focussing on ovulation risk and luteal progesterone levels in a given menstrual cycle, is associated with haemoglobin levels. The occurrence of ovulation is a clear binary signal of fecundity because it reflects either minimal investment in, or irrevocable suppression of, reproduction in that cycle. Luteal progesterone levels, predominantly driven by secretions from the corpus luteum, drive endometrial decidualisation and successful implantation. Whilst there is little available research on the optimum absolute or relative endogenous levels of progesterone required for successful implantation, ‘not enough’ is considered a barrier to successful pregnancy and is referred to in medicine by the ambiguous term ‘luteal phase deficiency (LPD)’, though this is a contested ‘disease’ [22]. Our study sample overcomes many of the hurdles involved in human trade-off research because (i) there was no modern contraceptive use and all study participants were married or in unions, and (ii) iron is a particularly important and limited resource in this population in which haemoglobin levels are normally high as an adaption to high-altitude hypoxia [23–25]. These conditions may allow us to observe trade-offs that are masked among individuals living in circumstances with lower iron requirements.

## 2. METHODS

### (a) Study population

The data for these analyses were originally collected as part of Project REPA (Reproduction and Ecology in Provincía Aroma), a longitudinal observational study among indigenous Aymara agropastoralists living in the Bolivian altiplano [26]. The data were collected from February to November 1996. These data were not collected to study the hypotheses tested here, therefore all data collectors and participants were blind to the present research questions. Women were from 30 rural communities scattered across approximately 400 km^2^, situated at an average of 3800 m altitude, approximately half-way between La Paz and Oruro. Families in these communities were predominantly reliant on potatoes, barley, sheep, and/or dairy cattle. The only formal medical care available was a small clinic in the local town of Patacamaya, but this is a substantial distance from most women’s homes. Inclusion criteria for Project REPA included falling within 20–40 years of age, currently in a stable heterosexual union, and not using contraception.

### (b) Ethics

All study protocols, including consent procedures, were approved by the Consejo Scientifico, Instituto Boliviano de Biologia de Altura, La Paz, and the Institutional Review Board, University of California, Riverside. All study participants were asked for verbal informed consent, which was obtained in accordance with the cultural norms of the study population. Giving, or declining to give, consent was recorded in confidential field records.

### (c) Analytical sample

The unit of analysis was a menstrual cycle, and cycles for which there were either hormone or anthropometric data were initially considered for analysis. This initial dataset comprised 817 menstrual cycles from 140 participants. As our focus was to investigate haemoglobin and fecundity, all cycles that were coded as pregnant or that did not have corresponding data for haemoglobin, ovulation, or progesterone were excluded from the analyses. Cycles with either a confirmed or possible early pregnancy loss (a positive hCG test followed by bleeding and a negative hCG test) were also excluded. Four cycles contained two measures of haemoglobin. For these cycles, the measurement taken latest in the cycle was retained in the dataset, as these cycles were often long and the latest measure was considered most likely to fall nearer to the putative ovulation date.

**Figure 1.**
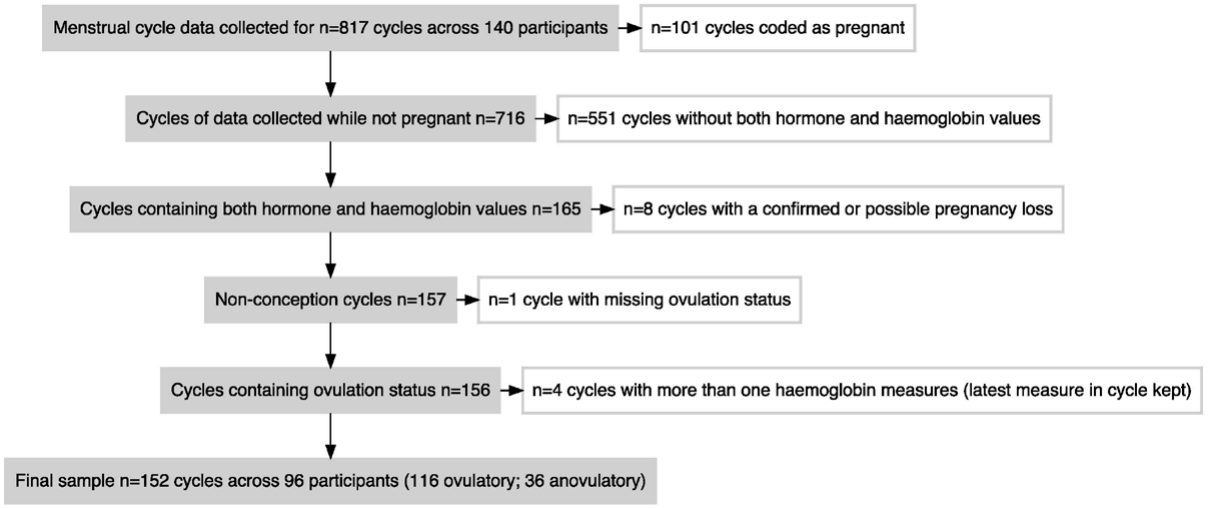
Sample creation flowchart

Miller’s (2016) review on the ecology of iron in women suggests different predictions for changes in haemoglobin in response to hormonal changes across an individual menstrual cycle [7]. For instance, haemoglobin may decrease with increased progesterone (due to increased fluid retention), but also may increase with increased oestrogen due to oestrogen’s blocking effect on hepcidin. An analysis of several variables of iron status across the menstrual cycle showed that whilst iron and hepcidin displayed a drop and rebound across menstruation followed by a stabilization in the second half of the cycle, haemoglobin levels did not consistently vary during the cycle [27]. We also found little evidence for variation in haemoglobin within menstrual cycles in our sample, and thus did not adjust for cycle day of the haemoglobin measurement. The final analytical sample consisted of 152 cycles from 96 participants (48 women contributed 1 cycle, 40 women contributed 2 cycles and 8 women contributed 3 cycles). The flowchart (Fig. 1) displays this process of sample creation.

### (d) Treatment of missing values

Overall, around 3% of the data was missing, mostly for the economic strategy score. Analysis of complete cases only by dropping missing cases can introduce bias and lead to a substantial reduction of statistical power [28]. To avoid dropping these cases, imputation using random forest multiple imputation was conducted using the R package ‘missRanger’ [29], which combines random forest imputation with predictive mean matching. Prior to all analyses, we imputed 5 datasets, with a maximum of 10 iterations specified for each imputation. To provide the most information for making imputation predictions, imputation of missing data was calculated using a larger dataset (e.g., participants’ weight, height, age at first birth) in addition to those variables that directly address our study questions. Parameter estimates for all five datasets were then pooled to provide more accurate estimates using the pool function from the R package ‘mice’ [30]. This function combines estimates from repeated complete data analyses into a single set of estimates and standard errors.

### (e) Causal inference

Whilst causal inference is very difficult in observational studies, recently scholars have argued that there is merit in acknowledging our intention to investigate causality, making our assumptions and limitations clear, and not shying away from the causal goal of research [31]. In order to think through which factors may be potential confounders and which to include as adjustment variables to help reduce bias in our analyses [32], we created a DAG (Directed Acyclic Graph) of hypothesized causal relationships [33] (Supplementary Figure 1). Based on this process, we minimally adjust for age for our first question, and age, body fat, and breastfeeding for our second question.

### (f) Variables

Haemoglobin (g/dl) was measured using a HemoCue (Quest Diagnostics), a portable, battery-operated point-of-care testing device that requires only a single blood drop from a finger prick. This procedure was concurrent with the collection of anthropometrics, typically done once per cycle in each woman’s home at a day and time arranged with her. Due to the presence of four low haemoglobin outliers, this variable was capped in order to meet the assumption of a normal distribution in our outcome variable in our multilevel linear models [34]. Capping takes any outliers below the fifth (11.8 g/dl) and above the 95th (17.5 g/dl) centiles and recodes their value to those of the 5th or 95th centiles, respectively. This allowed us to maintain potentially important observations close to their actual values and model them without losing quality in our models.

Ovulation is a binary variable (each cycle coded as ovulatory or anovulatory). Without ovulation there is no possibility of further reproductive investment during that cycle. Ovulation was ascribed to a given cycle using a previously published algorithm that is based on mean peak luteal progesterone concentration during the mid-luteal phase [26, 35].

Mean peak luteal progesterone (P4) concentration (pmol/l): Women were visited every other day (Monday, Wednesday, Friday or Tuesday, Thursday, Saturday) across the study period to record menstrual status and collect a 5 ml saliva sample, later assayed for progesterone. Mean peak luteal P4 is defined as the mean daily concentration during the span of days ±2.5 days from the day of observed peak luteal progesterone (i.e. (area under the curve of P4 from *x* to *y*)/(*y* − *x*) where *x* to *y* is any span of days and P4 at any time is defined by linear interpolation of the observed hormone data) [35]. This calculation is typically a more accurate estimate of the highest concentration of luteal progesterone than methods that assume progesterone peaks on a pre-specified cycle day (e.g. 7 days before menstrual bleeding begins). Such an assumption may miss the true peak and underestimate mean peak concentrations due to the considerable inter-cycle variation in the timing of the luteal progesterone peak. As our progesterone variable was positively skewed, the log of progesterone concentration was used in our regression models to avoid violating model assumptions. Only progesterone concentrations in ovulatory cycles were included in our analyses of progesterone levels (anovulatory cycles lack the corpus luteum that produces progesterone; inclusion of anovulatory cycles biases analyses. Supplementary Materials 2 for further justification).

Season was represented by a binary variable of arduous and better seasons across the year. For many women in this population, there are large seasonal differences in food availability, workload, and pathogen exposure. Numbering the days of the year from 1 to 366 (1996 was a leap year), the most arduous seasons consist of harvesting (late rain, early harvest days 1–91) and planting (late-winter/early-spring, before the summer rains begin, days 245–282) periods, which are more physically demanding than better periods in winter (late harvest, days 92–136, and after harvest before planting begins, days 137–244) and summer (the rainy season when crops are growing, days 283–366). In arduous periods, food availability is relatively lower, labour demands are higher, and pathogen exposure increases. These seasonal timings are based on weather data and direct observation during the year of data collection [36]. The distinction between arduous and better seasons is relative; even during better seasons, life is demanding in these altiplano communities. Arduous seasons account for a shorter proportion of the year at 129 days total, and better seasons account for 237 days total.

Traditional economic score is a measure of each household’s subsistence strategy based on principal component analysis of several ethnographically salient socio-economic variables [37]. This factor loads heavily on the husband’s frequency of salaried labour (negatively) and on the frequency of agricultural sales by the husband (positively). This factor represents the widespread Andean economic strategy of obtaining income through agricultural sales. Higher household scores reflect sufficient excess agricultural production and sales in the household rather than having to rely on wage labour for income.

Age in years was included to account for proximity to menopause or menarche.

Body fat score is a factor score from a factor analysis on all anthropometric body measurements taken in a given cycle. The factor loaded heavily on all weight, skinfold, and circumference measures, but not height, and explained 58% of the variation in all anthropometrics. This allowed us to capture energetic reserves available to women at the time of haemoglobin measurement.

Breastfeeding status is a binary variable, included to account for the suppressive effects of breastfeeding on ovulation and progesterone. All women in our sample were menstruating, so those who were breastfeeding were not experiencing lactational amenorrhoea.

### (g) Statistical analysis

Descriptive analysis presents variation in variables of interest across better seasons and more arduous seasons. To investigate the relationships between socio-ecological factors, haemoglobin, ovulation, and mean peak luteal progesterone, analyses were conducted using multilevel regression models with random intercepts by ID to account for the non-independence of multiple cycles sampled from the same individual (*n* = 48 with one cycle, *n* = 48 with multiple cycles) in this dataset. This was carried out using the glmmTMB package (see http://cran.nexr.com/web/packages/glmmTMB/glmmTMB.pdf) in R. All continuous independent or adjustment variables were centred on the mean and scaled by dividing by the standard deviation before use in models to obtain comparable effect estimates. Residuals for each model were checked using the DHARMa residual diagnostics package for hierarchical models (https://cran.r-project.org/web/packages/DHARMa/vignettes/DHARMa.html). Models were run (i) for all cycles and (ii) separately for cycles taken in more arduous and better seasons to examine whether the relationships between our variables of interest may be obscured by heterogeneity in seasonal conditions.

## 3. RESULTS

### (a) Descriptive results

**Figure 2.**
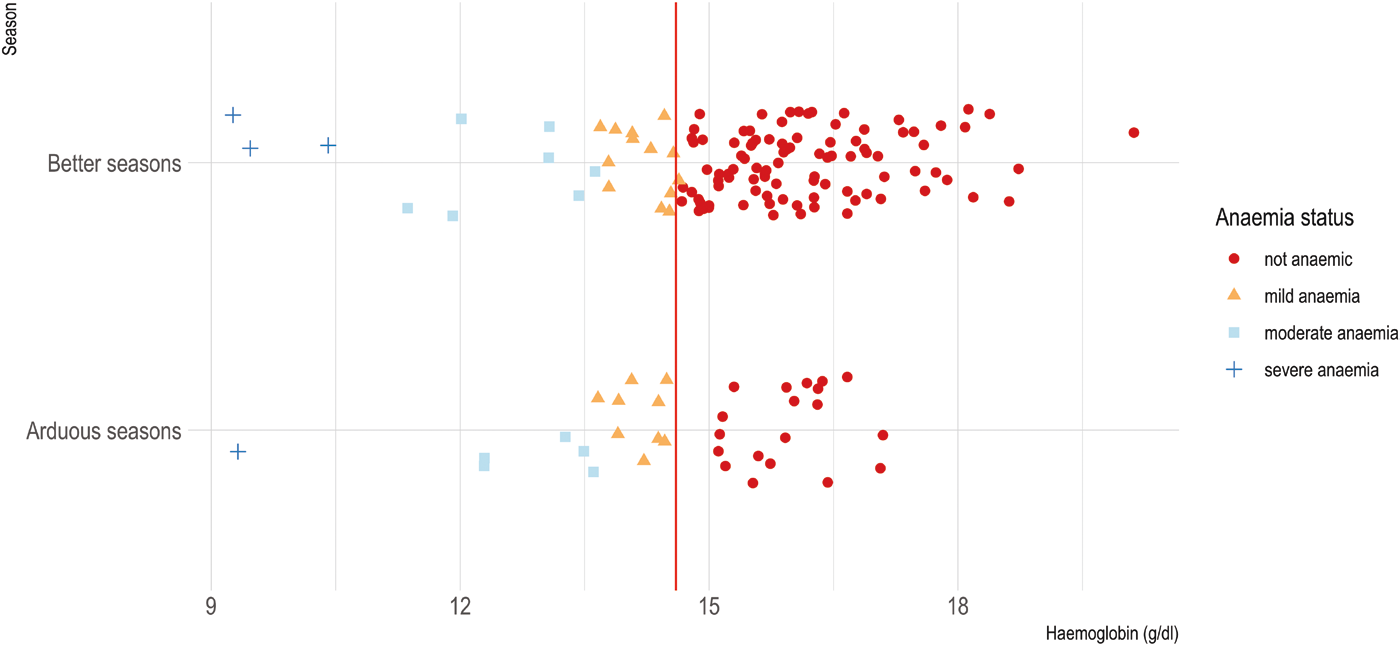
Distribution of haemoglobin values across seasons and anaemia status. The anaemia cut off here is 14.6 g/dl in keeping with the WHO recommendations to raise the anaemia threshold based on residence above sea level [38]

The median haemoglobin value was 15.6 g/dl with a range from 9.3 to 20.1 g/dl. Median haemoglobin was 15.15 g/dl in arduous seasons and 15.70 g/dl in better seasons (Table 1). When adjusting for altitude [38], over 25% of the sample was classified as anaemic (14% with mild anaemia, 8% with moderate anaemia and 3% with severe anaemia) (Fig. 2). In our sample, 71% of cycles in arduous seasons and 78% of cycles in better seasons were ovulatory, and there was a wide range of mean peak luteal progesterone concentration, with a median of 137 pmol/l and an interquartile range of 102–211 pmol/l in arduous seasons and a median of 182 pmol/l and an interquartile range of 130–260 pmol/l in better seasons.

**Table 1.** Descriptive statistics for all variables included in the analyses across seasons (*N* = 152 cycles total)

Variable	*N* cycles	Arduous seasons, *N* = 34^a^	Better seasons, *N* = 118^a^
Haemoglobin (g/dl)	152	15.15 (13.95, 15.97)	15.70 (14.90, 16.50)
Cycle type	152		
Anovulatory	36	10 (29%)	26 (22%)
Ovulatory	116	24 (71%)	92 (78%)
Mean peak luteal progesterone (pmol/l)	152	137 (102, 211)	182 (130, 260)
Traditional economic score	128	−0.22 (−1.15, 0.85)	−0.15 (−0.70, 0.57)
Age	152	31 (27, 34)	30 (24, 33)
Breastfeeding status	152		
Breastfeeding and menstruating	73	12 (35%)	61 (52%)
Menstruating	79	22 (65%)	57 (48%)
Time since last birth (days)	140	946 (546, 1,857)	612 (434, 1,112)
Body fat score	151	−0.11 (−1.24, 1.65)	0.92 (−1.16, 1.96)

^a^Median (IQR); *n* (%).

### (b) Are haemoglobin levels modulated by the socio-ecology?

**Figure 3.**
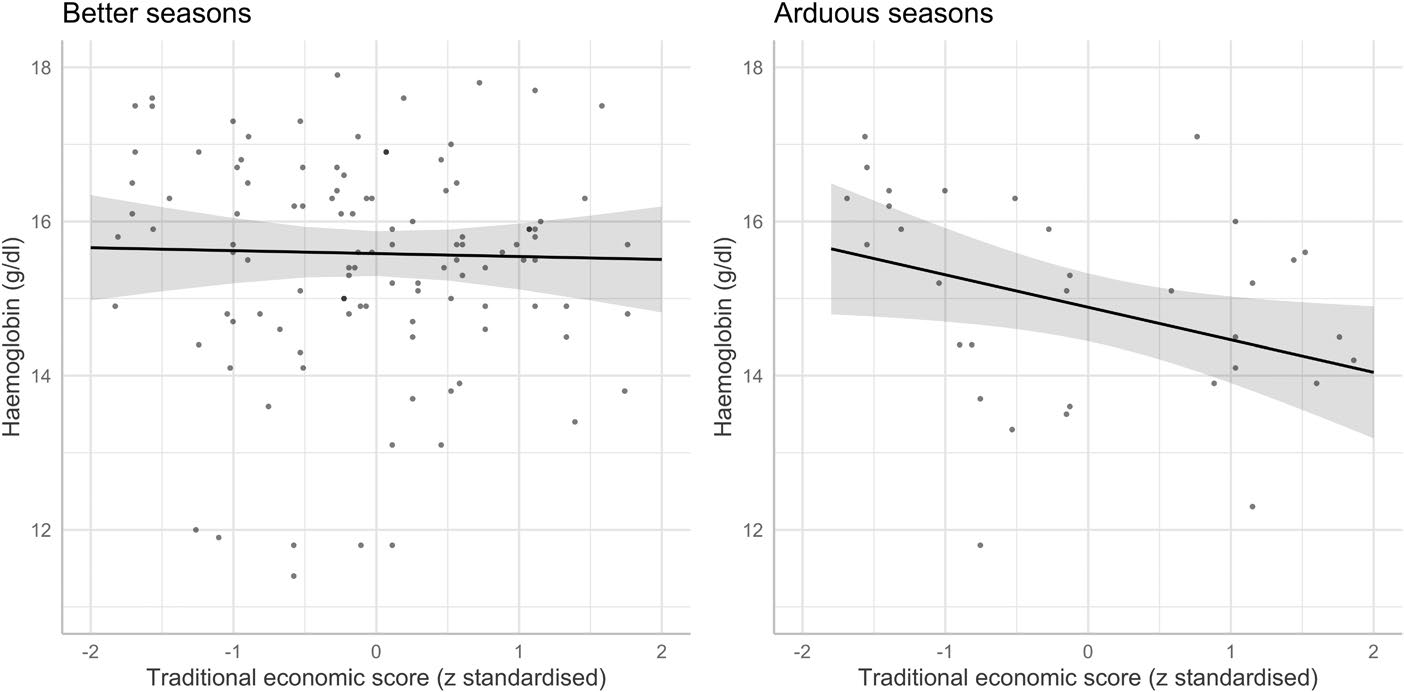
Predicted values for the age-adjusted relationship between traditional economic score and haemoglobin levels (g/dl) across seasons. Around half of the cycles are the only cycle for that woman and half of the women have multiple cycles. Shaded areas show 95% CIs

To investigate the relationship between socio-ecology and haemoglobin levels we used a linear mixed-effect model to account for repeated cycles within women. We found that on average haemoglobin levels were lower in the arduous seasons than in the better seasons (unadjusted β [95% CI] = 0.76 [0.24, 1.28], *P* = 0.005) with a predicted average of 14.85 g/dl in the arduous seasons compared to 15.61 g/dl in the better seasons. Evidence for this association remained when adjusting for age (adjusted β [95% CI] = 0.74 [0.22, 1.27], *P* = 0.006). Further, we found that, in the more arduous seasons, a higher traditional economic score was associated with reduced levels of haemoglobin both independently and after adjusting for age (both *P* = 0.032) (Table 2, Fig. 3), suggesting that women living in households reliant on traditional agropastoralism had lower levels of haemoglobin in the more arduous seasons compared to those reliant on other forms of income, such as salaried labour. We found no relationship between traditional economic score and haemoglobin in the better seasons.

**Table 2.** Model output showing crude and age-adjusted estimates for the effect of z-standardized traditional economic scores on haemoglobin levels across seasons

Effect of traditional economic score on haemoglobin	Crude estimates			Age-adjusted estimates		
Sample	Estimate [95% CI]	Standard error	*P* value	Estimate [95% CI]	Standard error	*P* value
All cycles (*N* = 152)	−0.16 [−0.42, 0.10]	0.13	0.235	−0.17 [−0.43, 0.09]	0.13	0.193
Better season (*N* = 118)	−0.02 [−0.33, 0.29]	0.16	0.881	−0.04 [−0.35, 0.27]	0.16	0.810
Arduous season (*N* = 34)	−**0.42 [**−**0.80,** −**0.04]**	**0.20**	**0.032**	−**0.42 [**−**0.81,** −**0.04]**	**0.20**	**0.032**

Bold values indicate *P* <0.05.

### (c) Is fecundity modulated by haemoglobin levels?

#### (i) Ovulation

**Figure 4.**
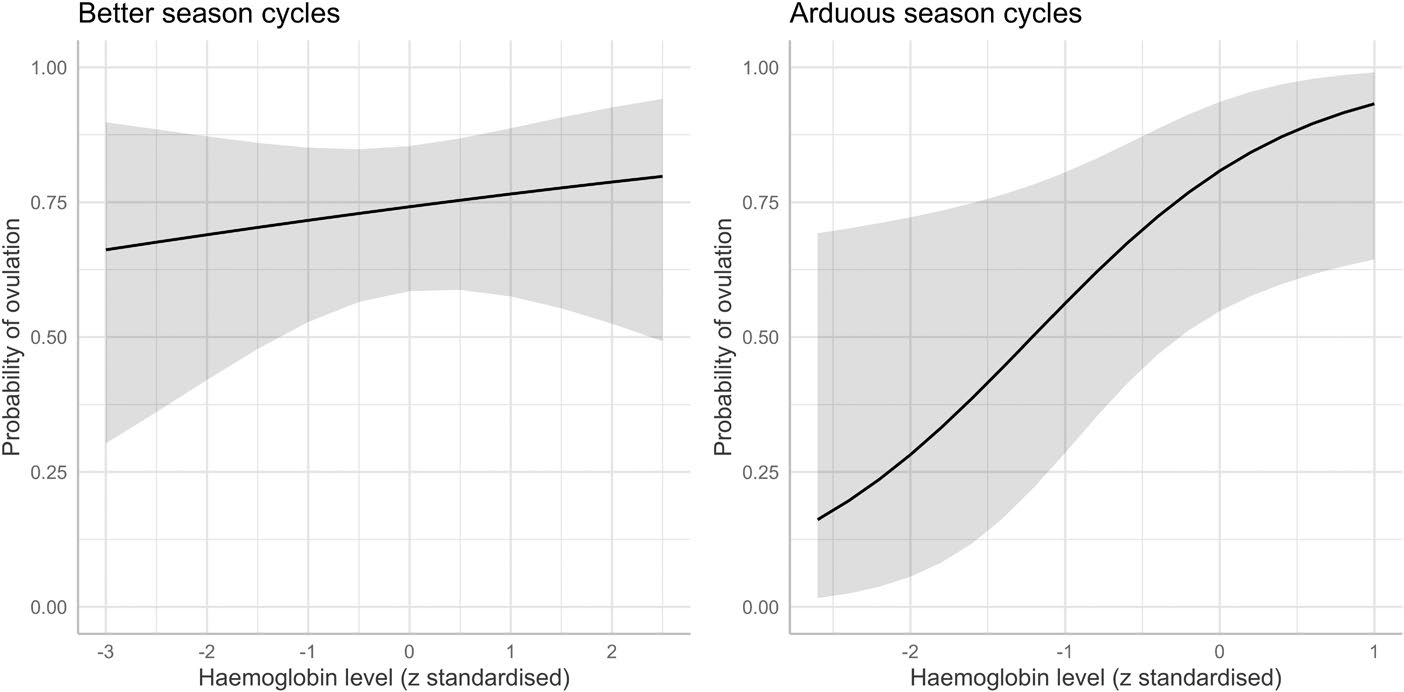
Predicted probabilities of ovulation across haemoglobin levels (z standardized) for cycles during the better and more arduous seasons and adjusted for age and breastfeeding status. Shaded areas show 95% CIs

To investigate the relationship between haemoglobin levels and the risk of ovulation, we used a mixed-effect logistic regression model. We found evidence that a one standard deviation increase in haemoglobin levels was associated with around three times the odds of ovulating in a given cycle during the more arduous seasons, both independently (OR = 2.59, 95% CI: [1.03, 6.54], *P* = 0.043) and when adjusting for age, body fat, and breastfeeding status (OR = 3.27, 95% CI [1.10, 9.77], *P* = 0.033). We found no relationship between ovulation and haemoglobin levels when including all cycles or cycles occurring during the better seasons (Table 3, Fig. 4).

**Table 3.** Crude and adjusted odds ratios for ovulation with a 1 standard deviation increase in haemoglobin

Effect of haemoglobin on ovulation	Crude odds ratios			Adjusted odds ratios^a^		
Sample	OR [95% CI]	Estimate standard error	*P* value	OR [95% CI]	Estimate standard error	*P* value
All cycles (*N* = 152)	1.40 [0.96, 2.03]	0.19	0.078	1.42 [0.97, 2.09]	0.20	0.074
Better season (*N* = 118)	1.17 [0.74, 1.84]	0.23	0.499	1.14 [0.72, 1.80]	0.24	0.588
Arduous season (*N* = 34)	**2.59 [1.03, 6.54]**	**0.47**	**0.043**	**3.27 [1.10, 9.77]**	**0.56**	**0.033**

Bold values indicate *P* <0.05.

^a^Adjusted for age, body fat, and breastfeeding status.

#### (ii) Mean peak luteal progesterone

To investigate the relationship between haemoglobin and mean peak luteal progesterone, we used a mixed-effect linear model. Mean peak luteal progesterone was not associated with haemoglobin in ovulatory cycles (Table 4). When adjusting for age, body fat, and breastfeeding status, there was a marginal weak positive relationship between progesterone and haemoglobin in the better seasons with an effect size of 0.08 (*P* = 0.054), but otherwise there was little evidence to suggest any relationship between haemoglobin and reproductive investment acting through progesterone levels.

**Table 4.** Crude and adjusted effect estimates for log-transformed mean peak luteal progesterone with a 1 standard deviation increase in haemoglobin in ovulatory cycles

Effect of haemoglobin on progesterone	Crude estimates			Adjusted estimates^a^		
Sample	Estimate [95% CI]	Standard error	*P* value	Estimate [95% CI]	Standard error	*P* value
All cycles (*N* = 152)	0.05 [−0.03, 0.12]	0.04	0.223	0.06 [−0.01, 0.13]	0.04	0.111
Better season (*N* = 118)	0.06 [−0.01, 0.14]	0.04	0.103	0.08 [0.00, 0.15]	0.04	0.054
Arduous season (*N* = 34)	−0.14 [−0.33, 0.05]	0.10	0.144	−0.12 [−0.29, 0.04]	0.09	0.146

^a^Adjusted for age, body fat, and breastfeeding status.

## 4. DISCUSSION

This study aimed to investigate the associations between iron status, as measured by haemoglobin levels, and markers of fecundity in a non-contracepting high fertility population from the Bolivian altiplano. We found that (i) haemoglobin levels were lower in arduous compared to better seasons; (ii) in arduous seasons, women in households with higher reliance on agropastoral subsistence strategies had lower haemoglobin levels compared to women in households more reliant on income from salaried labour; (iii) menstrual cycles with lower haemoglobin levels were more likely to be anovulatory during arduous seasons, independently of body fat; and (iv) among ovulatory cycles, haemoglobin levels were not associated with mean peak luteal progesterone. The results have implications for understanding how iron influences the regulation of natural fluctuations in reproductive capabilities.

The connection between haemoglobin levels, season, and traditional modes of subsistence could be influenced by several mechanisms including diminished iron stores (iron-deficiency anaemia) and/or anaemia caused by inflammation. For women reliant on agropastoralism, arduous seasons are characterized by high physical workloads, mostly done with hand tools, and typically involving travelling long distances to the fields for planting and harvesting. There is also reduced food availability and reduced availability of funds to purchase food elsewhere. Most of the time, meat is consumed only rarely and in small portions as part of a soup comprising potatoes, rice, a few vegetables, and occasionally quinoa. Dietary intake of iron is very low, particularly in the arduous seasons, and thus, the low haemoglobin levels observed during these periods may indeed represent depleted iron stores [39].

Even if iron stores are sufficient to avoid negative birth outcomes, the body may restrict how much bioavailable iron is circulating in the form of haemoglobin during arduous seasons to mitigate infection [40] when exposure is highest and suppress ovulation to avoid conception during times of low bioavailable iron for pregnancy, favouring investment in immunity over reproductive function. Pathogens need iron to survive and reproduce [41]. For instance, iron is crucial to the life cycle of tuberculosis mycobacteria [42] and an increase in dietary iron has been associated with increased chance of having active pulmonary tuberculosis [43]. In the population under study, exposure to infections is increased during arduous seasons with relatively isolated homesteads coming together for planting and harvesting with increased transmission of any pre-existing infections, weakened immune systems due to taxing workloads, and heighted exposure to infections in the environment, such as waterborne parasites. Risk of infection with tuberculosis and human fascioliasis increases during these seasons [44–47], potentially leading to inflammation-driven reduced iron absorption and reduced release of iron stores, and subsequently lower haemoglobin levels. In this context, restricting the level of bioavailable iron via the hormone hepcidin [2] can be adaptive as it reduces an infection’s ability to thrive. To pick apart the role of immune activity in determining haemoglobin levels, future studies should measure both an individual’s iron stores, through measuring serum ferritin or soluble transferrin receptor (sTfR), and markers of acute phase inflammation, such as C-reactive protein (CRP), for detecting the presence of infections.

Our results suggest that haemoglobin levels play a role in the modulation of fecundity, and this effect is independent of energetic stores. Whether driven by reduced iron stores or simply reduced iron availability, in the arduous seasons the risk of ovulation is reduced in the face of low haemoglobin levels. Whilst it might be assumed that women with higher haemoglobin levels may also have improved access to higher quality diets and thus greater energetic availability, instead we find that when adjusting for body fat score, a proxy for energetic status, an independent association between haemoglobin and ovulation remains in the arduous seasons. Both body fat and haemoglobin were positively and independently related to ovulation and as such, each can be considered necessary, but not sufficient, for ovulation to occur. Our findings support calls to focus on resources beyond energy for understanding mechanisms underlying evolutionary and physiological trade-offs [18, 48].

Our findings on the positive association between haemoglobin levels and ovulation risk in arduous but not in better seasons support the idea that organisms have evolved the capability to strategically allocate resources toward present or future reproduction based on the prevailing local socio-ecological conditions. According to the Flexible Response Model of female reproductive function [49, 50], natural selection favours organisms who are able to predict the best conditions they are likely to face in a given environment and, in such conditions, invest in reproduction rather than forego ovulation, even if said conditions are somewhat poor in absolute terms. Reduced investment in current reproduction may help avoid negative pregnancy outcomes [6] until iron stores are repleted, such as in better seasons when haemoglobin levels improve for women relying on agropastoralism. However, if haemoglobin levels are still low in the better seasons, there is reduced benefit to delaying reproduction further given that conditions are unlikely to improve. We include an exploration of whether women in our sample with low haemoglobin predictively suppressed ovulation at times where haemoglobin would subsequently increase in Supplementary Materials 3. This analysis is not included here as it is substantially limited by low power, but the direction of effect is supportive of women flexibly delaying ovulation when haemoglobin is low but will improve, and provides a springboard for future research.

Unlike for ovulation, we find little evidence to suggest that haemoglobin is associated with variation in mean peak luteal progesterone levels in ovulatory cycles. Firstly, this may be due to the index of progesterone we used (mean peak luteal progesterone), which reflects the absolute level of progesterone on and about the peak production of this hormone but does not reflect the magnitude of change from levels immediately after ovulation to the levels occurring at peak production. Recent research has suggested that *change* may actually be more biologically salient than *level* itself [18]. Secondly, we may fail to see a relationship due to sampling error as progesterone release is triggered in a pulsatile manner with wide variation in levels observed across the day depending on proximity to the pulsatile signal [51]. Finally, it could be that haemoglobin is not very relevant to the modulation of progesterone levels and the subsequent decidualisation of the endometrium and/or that progesterone levels above a necessary minimum are not essential to successful continuation of pregnancy during the weeks before the shift from luteal to predominantly placental production of progesterone [22, 26].

The main strength of our analysis lies in its ability to reveal human life history trade-offs that would otherwise not manifest or be statistically detectable, through the specificities of lives of Aymara women living at high-altitude in the Bolivian altiplano. Trade-offs are sometimes obscured by heterogeneity across a population or across time in access to resources or conditions experienced (Bolund, 2020). Our ability to analyse cycles collected from different seasons of the year among women utilizing different subsistence strategies allows us to investigate how trade-offs change over time and over conditions with the use of multilevel modelling to consider repeat measurements within the same women. We were limited in our ability to fully understand the kind of trade-offs we are observing and the mechanisms which underpin them without access to markers of infection, immune function, and iron stores. However, to collect extra markers in a large sample would have represented a significant extra cost and toll on participants, and highlights how studying the mechanisms underpinning trade-offs in humans is ethically and practically demanding [52]. Additionally, there are several potential selection biases which may have influenced our results. For instance, as we only included non-conceptive cycles, we have an overrepresentation of ‘non-successful’ cycles and so we cannot make inferences about the relationship between haemoglobin and ovulation in cycles that led to pregnancy.

Currently, it is recommended that iron supplements, typically in combination with folic acid, are given to general populations of women of reproductive age and pregnant women across the globe [53]. However, uncritical provision of supplementation without considering the trade-offs individuals face from a life history perspective has the potential to cause harm [54]. For instance, prenatal protein-energy supplementation has been shown to reduce subsequent birth intervals to the next child [55], with tightly spaced pregnancies taking a physical toll on women and, in some cases, returning the mother to a state of undernourishment. Given our findings, it is possible that iron supplementation of iron-deficient postpartum women, with the intention to raise iron stores and subsequent haemoglobin levels, could reduce the time to return of ovulation and shorten birth intervals with similar detrimental effects to protein-energy supplementation, particularly if the individual has been unable to replete other bodily reserves in time or is still breastfeeding. Additionally, if iron supplements are given in cases of non-iron-deficiency anaemia, most commonly driven by infection [56], this risks either being, at best, largely ineffective as iron gut absorption is heavily limited by hepcidin, or, at worst, puts women at a greater risk of illness if an infection is able to utilize the extra iron to proliferate [40]. Thus, it is crucial to contemplate iron supplementation only after evaluating a woman’s broader immune and reproductive circumstances and determining the specific type of anaemia she is experiencing.

In conclusion, our results among Aymara women living in the Bolivian altiplano support the hypothesis that fecundity is modulated based on iron status beyond energy intake. The potential for ovulation to be inhibited due to low haemoglobin is demonstrated, and this correlation exhibits a flexible response to the existing socio-economic factors and whether there is potential for seasonal improvement in conditions. Thus, we propose that iron status may be modulating resource allocation trade-offs between current and future reproduction and/or between immunity and reproduction and that these trade-offs should be considered when designing interventions to reduce anaemia.

## Supplementary Material

eoae022_suppl_Supplementary_Materials

## Data Availability

The data underlying this analysis is available via Dryad (access DOI: 10.5061/dryad.tqjq2bw7f).
