# Supplementary material for: Low haemoglobin in arduous seasons is associated with reduced chance of ovulation among women living in the Bolivian altiplano
Source: Evol Med Public Health. 2024 Sep 12;12(1):191–203. doi: 10.1093/emph/eoae022 (PMC11497615; doi:10.1093/emph/eoae022)
Supplement: eoae022_suppl_Supplementary_Materials [file eoae022_suppl_supplementary_materials.docx]

**Supplementary Materials**

**Figure S1 Directed Acyclic Graph (DAG) showing hypothesised causal relationships**

DAGs (Directed Acyclic Graphs) set out hypothesised causal relationships and help to think through which factors may be potential confounders to adjust for to reduce bias in our analyses. This DAG was created for investigating our two questions: Q1) Are haemoglobin levels modulated by environmental factors such as season and economic strategy? and Q2) Are measures of fecundity, such as ovulation and progesterone level, modulated by haemoglobin? For Q1, season and economic strategy are highlighted as the exposures (orange) and haemoglobin is highlighted as the outcome (green). For Q2, haemoglobin is highlighted as the exposure (green) and ovulation and progesterone highlighted as the outcomes (blue).

All variables used in our analyses are included as well as key latent unmeasured variables (e.g., infection and diet). Each arrow represents a hypothesised directional causal relationship and is based on relevant published literature. Using this DAG, we can identify that, to remove any confounding ‘back doors’, we must minimally adjust for age for our first question, and age, body fat, breastfeeding, diet, and infection for our second question. As diet and infection were not measured in our dataset, we were unable to adjust for these and must consider the risk of residual confounding of our estimates by these factors.

**S2 Modelling the relationship between haemoglobin and progesterone**

The decision was taken to run our analysis investigating the relationship between haemoglobin and mean peak luteal progesterone levels in ovulatory cycles only, rather than all cycles, for the following reasons:

1. From a modelling perspective, ovulation is highly associated with luteal progesterone levels (as can be seen in Figure S2 below). Therefore, as haemoglobin and ovulation are also associated with each other, any significant effect of haemoglobin on progesterone levels may be confounded by ovulation.

Indeed, when looking at the relationship between haemoglobin and luteal progesterone levels in all cycles (ovulatory and anovulatory), we do find some evidence for a positive relationship when adjusting for age, body fat, and breastfeeding status (β=0.09, 95% CI=0.00, 0.18, p=0.042). However, this effect does not remain when ovulation is added as an adjustment variable to the model (β=0.04, 95% CI=-0.02, 0.10, p=0.168).

**Figure S2 Relationship between haemoglobin level and mean peak luteal progesterone levels split by ovulation status.**

1. From a theoretical perspective, luteal progesterone levels are hypothesised to drive reproductive success through the preparation of the endometrium for successful implantation. This occurs through progesterone secretion from the corpus luteum after ovulation. If ovulation has not occurred, progesterone levels will not be driven by this mechanism and whether progesterone levels are high or low cannot contribute to successful reproduction, given that a chance at conception has already been forgone through anovulation. Therefore, conceptually, questions investigating reproductive modulation should exclude anovulatory cycles.

**S3 Exploratory analysis on predictive change over time**

In order to investigate whether delaying ovulation in the face of low haemoglobin levels during more arduous seasons could be considered a flexible response, predictive of future haemoglobin increases, we ran an additional exploratory analysis looking at haemoglobin change over time and ovulation. This analysis is limited by low power and as such is included here in supplementary materials as preliminary exploration of a direction of effect. However, the confidence intervals are extremely large and thus, should be interpreted with much caution and the results may best act as a springboard for future analyses to confirm or reject these trends.

To investigate the impact of change in haemoglobin on ovulation, we used a dataset with 1 row per two menstrual cycles to look at change from one cycle to the next measured (56 observations, comprised of 48 individuals with 2 cycles (contributing 1 row of dataset) and 8 individuals with 3 cycles (contributing 2 rows of dataset)). We did not use a multilevel model given that the small number of cycles contributed by the same person, which invalidates the assumptions of the multilevel models when the proportion that contribute more than one observation is so low.

We sought to investigate the hypothesis that if a woman was had low enough haemoglobin to be considered anaemic (<14.6g/dl) in her first cycle (cycle 1) and she went on to experience an increase in haemoglobin by her second cycle (cycle 2), then she would be less likely to ovulate in cycle 1, suggesting a delay in order to wait to ovulate when haemoglobin levels would be improved by cycle 2. This is predictive because the change in haemoglobin happens after cycle 1 has been ovulatory/anovulatory. Conversely, if haemoglobin decreases from cycle 1 to cycle 2 or remains the same, we predict that cycle 1 should be ovulatory as there is no benefit to delaying ovulation. Additionally, if a woman is not anaemic in cycle 1, she likely has enough haemoglobin to ovulate and reproduce successfully (assuming a threshold effect) and therefore we would not expect to see an effect of haemoglobin change on ovulation among those who already start with non-anaemic haemoglobin levels in cycle 1.

We modelled this question using ovulation in cycle 1 as our outcome variable with a logistic regression model. Change in haemoglobin from cycle 1 to cycle 2 in interaction with anaemia status in cycle 1 was our exposure variable. This relationship was modelled crudely and then minimally adjusted for age. We do not include season in the model because season is a strong predictor of haemoglobin level, and this is already contained in the model.

We find that the direction of effect from these models suggest that women who were anaemic (N=18) in their first cycle and who experience an increase in haemoglobin from their first to second cycle may be less likely to ovulate in their first cycle than those women who were not anaemic to start with (N=38) or who experienced no change or a decrease in haemoglobin from their first to second cycle (Table S3, Figure S3). Thus, if haemoglobin is likely to improve, women with low haemoglobin in the current cycle may be predictively delaying their ovulation until the next cycle. However, if haemoglobin is not likely to improve or is already sufficient, ovulation is more likely to occur in the current cycle. These trends suggest preliminary support for a predictive flexible response over time of ovulation to haemoglobin levels.

These trends should be interpreted with caution given our limited power and instead may act as food for thought for further future analyses. Based on Wald’s z-test for logistic regression coefficient power calculations, assuming a normal distribution of haemoglobin change between cycles and to detect change in probability of ovulation in cycle 1 of 10% with an alpha of 0.05, our anaemic sample of 18 women would have only a 26.5% chance of detecting an effect where one exists. To achieve 80% power, a sample of 73 anaemic women would be required.

**Table S1 Crude and age adjusted odds ratio estimates for a 1 standard deviation increase in the change in haemoglobin from cycle 1 to cycle 2 in interaction with anaemia status in cycle 1 (<14.6g/dl)**.

| *Effect of haemoglobin change and anaemia status on ovulation* | *Crude odds ratios* | | *Age adjusted odds ratios* | |
| --- | --- | --- | --- | --- |
| *Sample* | OR [95% CI] | p value | OR [95% CI] | p value |
| Haemoglobin change | 0.53 [0.12, 1.77] | 0.327 | 0.62 [0.13, 2.21] | 0.483 |
| Not anaemic in cycle 1 | 1.71 [0.29, 8.40] | 0.382 | 2.16 [0.34, 12.00] | 0.382 |
| Haemoglobin change * Not anaemic in cycle 1 | 1.74 [0.39, 9.70] | 0.487 | 1.63 [0.34, 9.93] | 0.558 |
| Age | - | - | **2.05 [1.04, 4.40]** | **0.047** |

**Figure S3 Predicted probability of ovulation in cycle 1 by anaemia status in cycle 1 (cut off 14.6g/dl) plotted against the z-standardised change in haemoglobin from cycle 1 to cycle 2 adjusted for age.**
